# Personalized dietary advices provided by a dietitian increase calcium intake in outpatients with multiple sclerosis—Results from a randomized, controlled, single-blind trial

**DOI:** 10.3389/fnut.2022.919336

**Published:** 2023-01-17

**Authors:** Sandrine Fiorella, Hanane Agherbi, Emilia El Houjeiry, Giovanni Castelnovo, Dimitri Renard, Pauline Privat, Elodie Santamaria, Virginie Vallayer, Sandrine Alonso, Thierry Chevallier, Candice Bancal, Sabine Laurent-Chabalier, Eric Thouvenot

**Affiliations:** ^1^Department of Neurology, CHU Nîmes, University of Montpellier, Montpellier, France; ^2^Unité Transversale de Nutrition Clinique, CHU Nîmes, University of Montpellier, Montpellier, France; ^3^Department of Biostatistics, Clinical Epidemiology, Public Health and Innovation in Methodology, CHU Nîmes, University of Montpellier, Montpellier, France; ^4^Laboratory of Biochemistry and Molecular Biology, CHU Nîmes, University of Montpellier, Montpellier, France; ^5^The Institute of Functional Genomics, University of Montpellier, CNRS, INSERM, Montpellier, France

**Keywords:** calcium intake, dietary advice, multiple sclerosis, coaching/accompaniment, personalized medicine

## Abstract

**Background and aims:**

Multiple sclerosis (MS) is associated with osteoporosis, possibly due to neurological disability and decreased calcium intake. The objective of this study was to evaluate the efficacy of a personalized nutritional advice program by a dietitian compared to the delivery of a standard advice form to optimize dietary calcium intake in outpatients with MS.

**Methods:**

We performed a randomized, controlled, parallel trial comparing the efficacy of a personalized dietary advice (PDA) program to standard advice form (SAF) to increase daily calcium intake in MS patients. The study population was composed by patients with relapsing-remitting MS aged 18–69 years old. PDA program consisted in dietary advice delivered by a dietitian at baseline, 1 month, and 3 months. Calcium and nutrient intake in patients from both groups was evaluated at baseline and 6 months using a dietary survey.

**Results:**

Of the 194 patients screened for inclusion, 182 patients were included (79% female, median age of 42 years, and median EDSS of 2.0), and randomized to SAF (*n* = 92) or PDA (*n* = 90). At 6 months, median calcium intake increased by 241 mg/day in the PDA group and decreased by 120 mg/day in the SAF group (*p* < 0.0001). However, the median calcium intake was 947 mg/day in the SAF group and 778 mg/day in the PDA group at baseline (*p* = 0.0077), potentially favoring the effect of dietary advice. Complementary analyses focusing on patients with insufficient calcium intakes at baseline revealed comparable values in both groups (*p* = 0.69). Of those, patients included in the PDA group obtained significantly higher calcium intakes at 6 months than patients from the SAF group (*p* = 0.0086) independently of EDSS, PASAT, HADS and EQ-5D scores.

**Conclusion:**

This work shows the efficacy of dietary management based on personalized advice program over 3 months to durably increase calcium consumption in MS patients with insufficient calcium intake.

**Clinical trial registration:**

clinicaltrials.gov, identifier NCT02664623.

## Introduction

Calcium is an essential nutrient playing a key role in skeletal mineralization, as well as a wide range of biologic functions (1). Prolonged insufficient calcium intake promotes the occurrence of osteoporosis or worsens ongoing osteoporosis, resulting in decreased bone mineral density (BMD) and disruption of bone micro-architecture, consequently increasing bone fragility and predisposing to a higher risk of bone fractures, most often of the wrist, vertebrae and femoral neck (2, 3). Osteoporosis can be prevented by adequate calcium and vitamin D intake (4, 5).

Multiple sclerosis (MS), a chronic, auto-immune, demyelinating disease of the central nervous system affecting mainly young women, is a multifactorial disease that appears to be influenced by genetic and environmental factors (6). MS is the leading non-traumatic cause of acquired severe disability in young patients (7). MS patients have a greater risk of osteoporosis than the general population as low BMD appears to occur in the early-stage of the disease (8–11), attributed to neurological disability, specific treatments, concomitant use of corticosteroids and lack of exposure to sunlight (25-hydroxyvitamin D—25OHD—deficiency). In addition, patients diagnosed with an auto-immune disease such as MS often choose to follow a gluten-free diet and reduce or avoid consuming dairy products, as these diets have been suggested to improve disease outcomes (12–15). In fact, the low-saturated fat (e.g., the so-called Swank diet) and Paleolithic diets have shown promise for MS symptoms, although inadequate calcium and vitamin D intake have been observed due to the restriction of specific foods (16, 17). Milk and dairy products on average provide 50–60% of daily calcium intake, so should be replaced by other calcium rich elements like certain mineral waters, canned fish such as sardines, and fruit and vegetables rich diet (18). With a regular dietary survey, an exogenous supplementation can compensate the calcium deficiency to reach the recommended dietary allowance (RDA) of calcium (18). MS is also associated with malnutrition in advanced disease stages because of increasing motor and cognitive disability that should be detected and treated (19, 20). Doing so, it is important to consider multiple factors like age, sex and education level that are known to influence diet quality and health behavior patterns (21–23).

We hypothesized that a dietitian, by proposing appropriate nutritional strategy, would contribute to optimize calcium intake in MS patients. We designed a randomized controlled trial in MS patients comparing the efficacy of a personalized dietary advice (PDA) program to standard advice form (SAF) to modify patient behavior and thus improve calcium intake. We also analyzed the impact of different conditions associated with MS (disability, cognitive status, anxiety, depression and quality of life) on these interventions.

## Materials and methods

### Study design and participants

This study was a randomized, controlled, single-blinded, parallel trial. The study population consisted of MS outpatients with Expanded Disability Status Scale (EDSS) score <6.5 followed in the departments of Neurology of Alès Hospital and University Hospital of Nîmes. Eligible patients were aged between 18 and 70 years with confirmed RRMS, without previous dietary consultation for calcium intake. Patients were excluded if they were unable to complete the self-questionnaire, if we identified at screening that they had vitamin D deficiency related to a history of progressive gastro-intestinal or systemic disorder, moderate renal impairment (creatinine clearance <60 ml/min) or if they were vulnerable to hypercalcemia (e.g., history of cardiac arrhythmia or disease, renal lithiasis, or undergoing treatment with digitalis drug products). Patients meeting the inclusion criteria were contacted by phone within 30 days before the next follow-up consultation by the neurologist to determine if they were interested in participating in the study conducted following their next neurology consultation. Interested patients were sent the study information leaflet and a dietary diary to complete by post. The study dietitian called the patient within 10 days prior to the visit to verify that the documents had been received and explained how to complete the dietary diary. All patients assessed in the study signed the consent form.

This study was conducted in compliance with law no. 2004-806 of 9 August 2004 relating to public health policy and to its application decisions, the declaration of Helsinki and Good Clinical Practice. The study received ethics approval by the French national agency for the safety of medicines and health products (ANSM) and by National Persons Protection Committee (CPP) Sud Méditerranée III (# 2015.11.01 ter) and was prospectively registered on clinicaltrials.gov (NCT02664623).

### Intervention

All patients were included by a neurologist during the neurology visit and randomized into two groups: personalized dietary advice (PDA) and standard advice form (SAF). They next attended a dietitian interview during which calcium, nutrient and energy intakes were evaluated over the last three days, coupled with an evaluation of food consumption habits (gluten free, lactose free, vegetarian, and other specific diets). These interviews were conducted according to the recommendations of the French Association of Dietitians Nutritionists (AFDN) (24). The patient’s meals of the last three days were entered into a survey calculation software (DATAMEAL) allowing the calculation of calcium and others nutrients intakes based on CIQUAL database (database from the ANSES website Agence Nationale de Sécurité Sanitaire de l’Alimentation, de l’Environnement et du Travail (ANSES), Ciqual (Table de composition nutritionnelle des aliments) (25). In the Ciqual database, energy, macronutrients (proteins, carbohydrates, lipids) and micronutrients (vitamins and minerals), among others, are indicated per 100g of food. The average intake was calculated over these 3 days and considered for the study. Then, all patients were delivered an advice form from GRIO (Groupe de Recherche et d’Information sur les Ostéoporoses) (26). This learned society, created 30 years ago by physicians and rehabilitation specialists, aims at educating care givers, informing the public and promoting research on medical bone pathologies, particularly osteoporosis. This document provided information to patients in both groups on how to improve calcium intakes. The GRIO form is a two-page document in French, indicating the optimal target for daily calcium intake and provides information on the calcium content of different food groups such as fruits, vegetables, calcium-rich mineral waters and other protein-rich foods (Supplementary Figure 1).

Patients randomized to the PDA group had an additional personalized interview at baseline with the dietitian lasting at least 20 min, to propose a healthcare plan with negotiated objectives appropriate for the patient’s situation, optimizing calcium intake and limiting risk of fractures. They aimed at optimizing calcium intake by increasing the consumption of calcium-rich foods compatible with the patient’s dietary profile (choice of dairy products, fatty fish such as sardines, calcium-rich mineral waters, vegetables, oleaginous fruits) and at limiting the risk of osteoporotic fractures (balanced diet sufficiently rich in proteins and adapted physical activity, limitation of overweight, etc.). They also attended two other consultations with the study dietitian at 1 and 3 months to enhance the motivational levers concerning the nutritional changes recommended at baseline. At this stage of the patient’s care, the qualitative evaluation of calcium intake (survey of consumption frequencies, not quantified) provided the professional with information on the implementation of the proposed advice, on the obstacles encountered and possible adjustments. Nutritional coaching were dedicated to refinement of calcium objectives, readjustment of calcium intake and reduction of osteoporosis risk factors (vitamin D deficiency, insufficient physical activity, low BMI, smoking, etc.). At the end of the M1 or M3 visits, patients from the PDA group received again the GRIO form annotated with personalized and readjusted advice. The same dietitian followed the patient in the PDA group during this study at baseline, M1 and M3, in order to reproduce the personalized educational management scheme.

Patients from both groups had a further neurology and dietitian consultation at 6 months with another dietitian blinded from randomization to evaluate calcium intake based on CIQUAL data using the same procedure that at baseline.

### Objectives

The primary objective of this study was to evaluate the change in calcium intake (mg/day) at 6 months from baseline between PDA and SAF groups, evaluated by a dietary survey.

The secondary objectives of the study were to examine the effect of PDA approach on 25OHD levels and to correlate these to calcium intake. We also evaluated the impact of MS conditions (disability, cognitive status, anxiety, depression and quality of life) on the efficacy of the interventions and the impact of diet modifications after MS diagnosis on calcium intake at baseline, as an indirect effect of the disease.

### Sample size

To determine the sample size, we relied on results of previous studies (27, 28). One study described a 12% increase in calcium intake 1 month after a therapeutic education program including a dietary consultation (27). We hypothesized that the increase would be higher in the PDA arm, approximately 20% at 3 months after three consultations with a dietitian. Based on another study, we assumed a baseline calcium intake of 917 ± 271 mg/day (28). So, hypothesizing a 5% increase in the SAF group (963 mg/day after the intervention, i.e., +27 mg/day) and 20% in the PDA group (1,100 mg/day after intervention, i.e., +183 mg/day), 82 patients per group were calculated to be necessary to demonstrate this difference with a power of 90% and a type 1 error alpha of 5%. Anticipating 10% unevaluable data, 91 patients would be needed per group for a total of 182 patients (the common standard deviation of the difference has been set at 271).

### Stratification

Calcium recommended dietary allowances (RDA) by the ANSES in 2016 (ANSES 2016) were 1200 mg/day for men over 65 years old and postmenopausal women aged over 51 years old, and 900 mg/day for other adults (29). We defined three classes of patients according to these recommendations (Supplementary Table 1): (1) the InfraRDA for patients with insufficient daily calcium intake (<900 mg/day for men >65 years and postmenopausal women >51 years and <750 mg/day for other patients); (2) the SubOptiRDA for patients whose calcium intake is close to the recommendation (between 900 and 1,200 mg/day for men >65 years and postmenopausal women >51 years, and between 750 and 900 mg/day for other patients); (3) the SupraRDA for patients with a calcium intake above the recommendations (>1,200 mg/day for men >65 years and postmenopausal women >51 years and >900 mg/day for other patients). According to this classification, we first stratified baseline population for analysis of calcium intake change over 6 months. At 6 months, patients were classified again to examine potential class change during follow-up in each intervention group. As ANSES recommendations (ANSES 2019) changed during the study (Supplementary Table 1), we also performed an ancillary analysis using these new recommendations (30).

### Randomization and blinding

After checking the eligibility criteria, patients were randomly assigned (1:1) to PDA group or SAF group according to a randomization list stratified on the center. This list was established by an independent statistician of CHU de Nîmes using specifically designed SAS (Cary, NC, USA) program. Only this statistician knew the number of subjects by block size of 4 or 6. Random allocation sequence was centralized to an online application to which recruiting investigators had access via connection with personal login and password. An independent dietitian evaluating calcium intake at 6 months was blinded to group assignment. Blinding was not possible for patient and study dietitian.

### Data collection

During the neurology visit at baseline and 6 months, all patients underwent neurological disability evaluation according to the EDSS (31), scoring disability according to eight systems (bowel and bladder, vision, mental and other) or functional parameters (pyramidal function, cerebellar function, sensory function, and brain stem function). Patients’ age, sex and education level [low education level (LEL) < school diploma ≤ high Education level (HEL)] were collected at baseline. Calcium and nutrient intake were evaluated at baseline and at 6 months by a dietary survey calculated using the nutritional composition of foods described in the CIQUAL database (Consulted between 13 July 2016 to 1 January 2020) (25). The Paced Auditory Serial Addition Test (PASAT) (32) was used to evaluate the ability to process information, assess cognitive status and measure sustained and divided attention. The Hospital Anxiety and Depression Scale questionnaire (HADS) (33) validated in French (34) was used to assess anxiety and depressive disorders. It contains seven questions each on anxiety and depression. The final score classifies anxiety and depression symptoms as follows, 0–7: normal; 8–10: average; 11–14: moderate; 15–21: severe; and we considered patients with a score <11 as having no or low disorder and those with a score ≥11 as having a serious disorder (moderate or severe). Quality of life was evaluated by the EQ-5D (35), the total score of the questionnaire is a utility value calculated in relation to the “France” reference with the tool available on the EuroQOL website (36). Five dimensions are measured (mobility, self-sufficiency, routine activities, pain/discomfort, anxiety/depression), on a 5-point Likert scale (“no problem” to “extreme problems”). The responses can be combined in a 5-digit number describing the patient’s health status. We chose a cut off at 0.7 (median score of the study population) and we evaluated patient’s quality of life as follows: bad < 0.7 ≤ good. Blood samples from participants were collected at baseline and 6 months. Circulating levels of 25OHD (concentration in nmol/L) were determined from EDTA serum samples with the Elecsys vitamin D total II kit using the cobas e 801 analytical unit (Roche Diagnostics, USA). This assay reflects vitamin D2 and vitamin D3 sources, although the majority is vitamin D3 given the very limited sources of vitamin D2 unless a participant is taking a vitamin D2 supplement. The laboratory is certified for French quality standards NF ISO15189 and ISO22870. The intra-assay precision was 5.8% for low-level (27.28 nmol/L) and 3.1% for medium level (78.95 nmol/L) measures. The inter-assay precision was 6.56% for low-level measure (27.28 nmol/L) and 5.36% for medium level (78.95 nmol/L). For 2020, the bias (long term) was 0.2%.

### Data analysis

Categorical variables are expressed as counts, percentages, and continuous variables as medians and interquartile ranges (IQR) because of their distribution. Baseline characteristic population and calcium intakes between SAF vs. PDA groups were compared using the chi-square or Fisher’s exact test for qualitative variables and the Wilcoxon-Mann-Whitney test for quantitative variables. Spearman r was used for correlation between calcium intakes and levels of 25OHD. Wilcoxon-Mann–Whitney test have been used to compare calcium intake between SAF and PDA subgroups (InfraRDA, subOptiRDA, and SupraRDA) after stratification. Holm and false discovery rate (FDR) correction were used for multiple comparisons. Group effect on 6 months calcium intake was analyzed with adjustment factors using linear model. Analyses were performed with a bilateral alpha level of 0.05 using SAS software, version 9.4 (SAS Institute, Cary, NC, USA).

## Results

**FIGURE 1:**
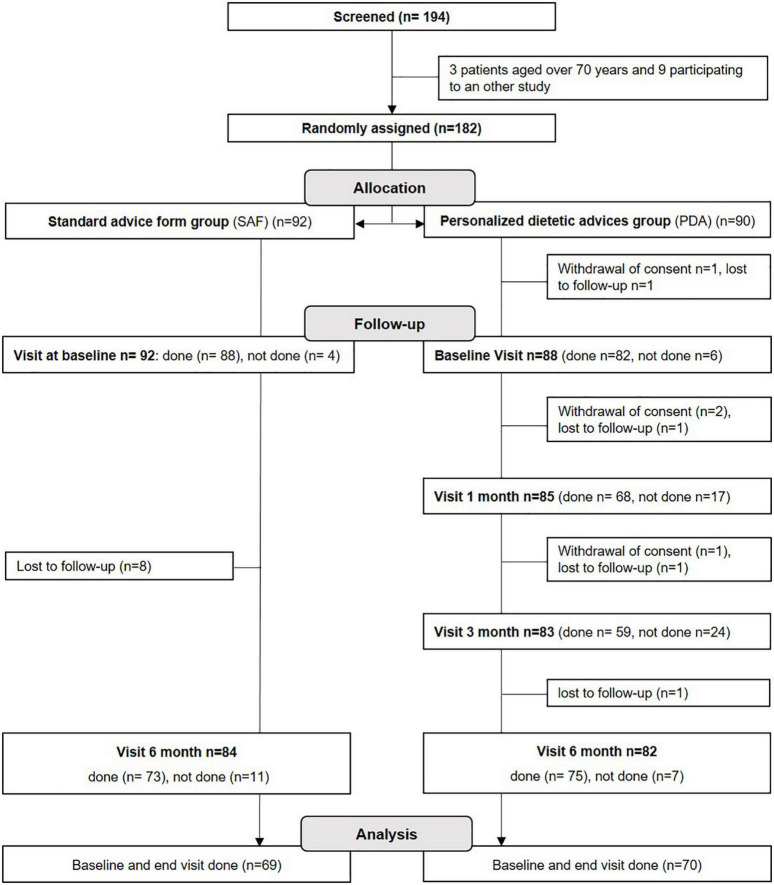
Study flow chart.

The flowchart of the study is shown in Figure 1. Out of the 194 patients screened for the study, 182 were recruited between July 2016 and April 2019. Final follow-up visits were completed by October 2019. Ninety-two patients were assigned to the SAF group and 90 to the PDA group. Three patients withdrew consent, 12 patients were lost to follow-up and 28 patients did not attend the baseline or the 6 month visit. Altogether, data from 139 patients who completed the study were analyzed (*n* = 69 from the SAF group and *n* = 70 from the PDA group). Analysis of serious adverse events during the study showed that the safety was good as only 10 serious adverse events were reported and none of them were related to the interventions. Serious adverse events occurring are related to an independent medical examination (*n* = 1, lymph-node biopsy), to surgical and medical procedures (*n* = 6, 2 induced abortions, 1 cesarean procedure for fetal cardiac rhythm disorder at 30 weeks of amenorrhea, 1 cholecystectomy, 1 sinus surgery by endoscopy, 1 case of botulinum toxin injection for the treatment of overactive bladder syndrome), to gastro-intestinal disorders (*n* = 2, 1 subileus and 1 intestinal perforation), or to MS (1 case of vertigo and nausea attributed to MS) (data not shown).

### Baseline characteristics

Baseline demographic and clinical parameters of the entire population (*n* = 182) are shown in Table 1. Median age was 42.0, 79% were females, median disease duration was 6 years and median BMI was 23.2. Evaluation of neurological disability and cognitive impairment revealed a median EDSS of 2.0 and a median PASAT of 39.0. Median daily lipid, carbohydrate and protein intake was 74.5, 181, and 73 g, respectively. Median daily calcium intake was 865.5 mg, vitamin D consumption 2.0 μg (Table 1), and median serum level of 25OHD was 62 nmol/L. Calcium intake at inclusion was not correlated with age, disease duration, EDSS, PASAT, and serum levels of 25OHD (Spearman *r* = 0.02, −0.04, −0.11, 0.18, and 0.011, respectively). Five patients (including 4 PDA and 1 SAF patients) reported calcium supplementation and 33 patients (including 20 PDA and 13 SAF patients) reported Vitamin D supplementation at baseline. Despite randomization, baseline calcium intake in the SAF group was higher (947 mg/day) than in the PDA group (778 mg/day, *p* = 0.0077).

**TABLE 1:** Baseline characteristics.

Variable	Total (*n* = 182)		SAF (*n* = 92)		PDA (*n* = 90)		*P*-value
Sex, male/female	182	39 (21.4%)/143 (78.6%)	92	18 (19.6%)/74 (80.4%)	90	21 (23.3%)/69 (76.7%)	0.5356^a^
Education level, (LEL/HEL)	180	69 (38.3%)/111 (61.7%)	90	36 (40%)/54 (60%)	90	33 (36.7%)/57 (63.3%)	0.6456^a^
Age, years	182	42 [34; 49]	92	39.5 [33; 46.5]	90	43 [34; 53]	**0.0351** ^b^
Weight, kg	167	65 [57; 79]	86	66.5 [58; 82]	81	65 [56; 78]	0.3801^b^
Height, cm	180	166 [160.5; 173]	91	166 [162; 173]	89	167 [160; 173]	0.7203^b^
BMI, kg/m^2^	167	23.2 [20.8; 28.7]	86	23.4 [21.3; 28.7]	81	22.9 [20.7; 28.5]	0.6253^b^
Calcium intake, mg	170	865.5 [612; 1095]	88	948 [764.5; 1149.5]	82	772 [579; 941]	**0.0027** ^b^
Proteins, g	170	73 [58; 93]	88	76 [62.5; 100]	82	67.5 [57; 89]	**0.0231** ^b^
Lipids, g	170	74.5 [57; 98]	88	78 [61; 110]	82	72.5 [55; 91]	0.1081^b^
Carbohydrates, g	170	181 [134; 240]	88	179.5 [137; 245.5]	82	182.5 [131; 222]	0.8275^b^
Energy, kCa	170	1767 [1411; 2191]	88	1827.5 [1448.5; 2255.5]	82	1744.5 [1349; 2049]	0.1764^b^
Vitamin D (μg)	170	2.0 [1.3; 3]	88	2.0 [1.5; 3.8]	82	2.0 [1.1; 2.4]	**0.0100** ^b^
25OHD (nmol/L)	172	62 [45; 90]	86	60 [41; 87]	87	64 [47; 95]	0.3497^b^
PASAT score	152	39 [30.5; 49]	78	38.5 [31; 49]	74	39 [29; 46]	0.8453^b^
EDSS score	181	2.0 [1; 4]	92	1.5 [1; 4]	89	2.0 [1; 4]	0.3929^b^
MS duration (years)	182	6 [3;14]	92	5 [2; 12]	90	7 [4; 16]	**0.0176** ^b^

SAF, standard advice form approach; PDA, personalized dietary advice approach; LEL, low education level; HEL, high education level; PASAT, Paced Auditory Serial Addition Test; EDSS, Expanded Disability Status Scale.

Data presented as number (%) or median [q1;q3]. *P*-values in bold denote significant differences.

^a^Statistical significance between groups was calculated by Chi-square test.

^b^Statistical significance between groups was calculated by Wilcoxon–Mann–Whitney test.

### Evolution of calcium intake at 6 months

**FIGURE 2:**
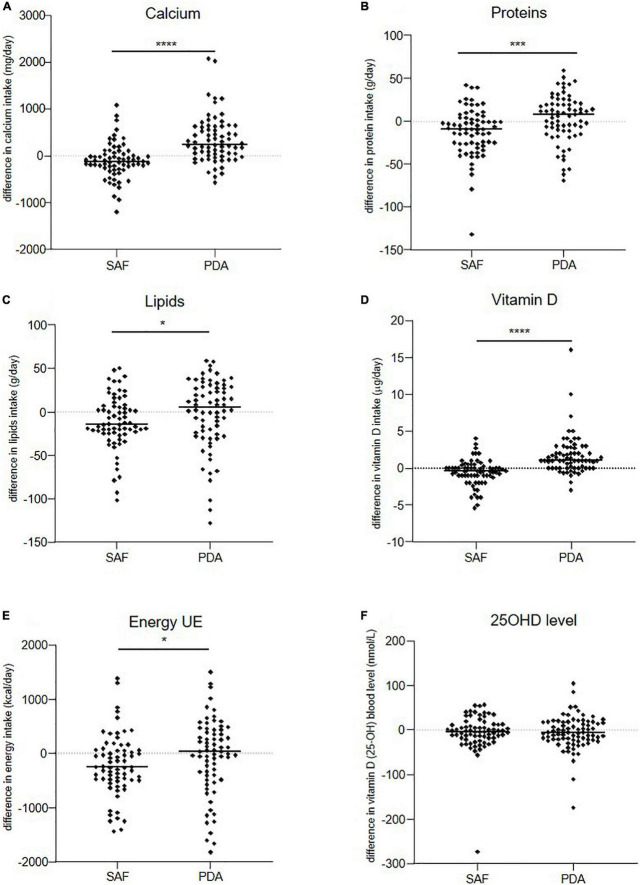
Evolution over 6 months of patients’ daily intakes. **(A)** Calcium, **(B)** proteins, **(C)** lipids, **(D)** vitamin D, **(E)** energy, and **(F)** level of serum 25OHD in SAF and PDA groups. Means are shown as horizontal bars. Statistical significance was determined using the Wilcoxon–Mann–Whitney test. *p < 0.05, ***p < 0.001, ****p < 0.0001.

Over the 6-month follow-up period, 139 participants were analyzed, 9 patients reported calcium supplementation (including 6 PDA and 3 SAF patients) and 51 patients (29 PDA and 22 SAF patients) reported Vitamin D supplementation at baseline. The median calcium intake for the SAF group was 816 mg vs. 1016.5 mg for the PDA group (*p* = 0.0004) showing at 6 months, a significantly greater increase in median calcium intake in the PDA group (+241 mg/day) than in the SAF group (−120 mg/day) (*p* < 0.0001) (Table 2 and Figure 2A). This significant group effect was confirmed by an analysis with adjusted linear model on baseline calcium intake (*p* < 0.0001). To complete our analysis, we stratified, in each group, the baseline population according to the recommendations of ANSES 2016 and focused on InfraRDA patients with insufficient calcium intake (Supplementary Tables 1, 2). Among the 139 patients who completed the study, 52 patients were InfraRDA (17 in the SAF group and 35 in the PDA group), 26 patients were SubOptiRDA (16 in the SAF group and 10 in the PDA group) and 61 patients were SupraRDA (36 in the SAF group and 25 in the PDA group) at baseline (Table 3).

**TABLE 2:** Calcium intake of total and stratified baseline population in PDA and SAF groups and evolution of calcium intake at 6 months in each subgroup.

	Baseline					At 6 months				
	SAF		PDA		*P*-value	SAF		PDA		*P*-value
Total population	69	947 [760; 1,150]	70	778 [583; 941]	**0.0077**	69	816 [617; 1,023]	70	1,016.5 [822; 1,391]	**0.0004**
InfraRDA	17	578 [494; 668]	35	583 [497; 650]	0.6909	17	617 [525; 704]	35	939 [791; 1,067]	**0.0086**
SubOptiRDA	16	837.5 [790; 896.5]	10	812 [790; 844]	0.4363	16	817.5 [680.5; 946]	10	932.5 [787; 1,218]	0.227
SupraRDA	36	1,149.5 [1,027; 1,431.5]	25	1,169 [940; 1,340]	0.5165	36	945 [753.5; 1,068]	25	1,283 [1,059; 1,765]	**0.0007**

Daily calcium intake is presented as median [q1; q3]. *P*-values in bold denote significant differences.

Statistical significance between groups was calculated by Wilcoxon–Mann–Whitney test.

SAF, standard advice form approach; PDA, personalized dietary advice approach; InfraRDA, baseline patients with insufficient daily calcium intake (<900 mg/day for men 65+ years old and postmenopausal women 51+ years old and <750 mg/day for others); SubOptiRDA, baseline patients whose calcium intake is close to the recommendation (900–1,200 mg/day for men 65+ years old and postmenopausal women 51+ years old, and 750–900 mg/day for others); SupraRDA, baseline patients with a calcium intake above the recommendations (>1,200 mg/day for men 65+ years old and postmenopausal women 51+ years old with a daily calcium intake and >900 mg/day for others).

**TABLE 3:** Daily calcium intake evolution over 6 months in PDA and SAF groups.

	Calcium intake evolution at 6 months				
	SAF		PDA		
Group	***N***	**Median** **IQR**[**q1;q3**]	***N***	**Median** **IQR**[**q1;q3**]	***P*-value**
Total population	**69**	**−120** [**−257; 42**]	**70**	**241.5** [**7; 629**]	**<0.0001**
InfraRDA	**17**	**66** [**−45; 251**]	**35**	**358** [**172; 631**]	**0.0082**
SubOptiRDA	**16**	**−78** [**−144.5; 108.5**]	**10**	**120.5** [**−84; 436**]	0.1389
SupraRDA	**36**	**−232** [**−513.5; −140**]	**25**	**124** [**−92; 540**]	**0.0004**

Data presented as median [q1; q3]. *P*-values in bold denote significant differences. Statistical significance between groups was calculated by Wilcoxon–Mann–Whitney test. SAF, standard advice form approach; PDA, personalized dietary advice approach.

**FIGURE 3:**
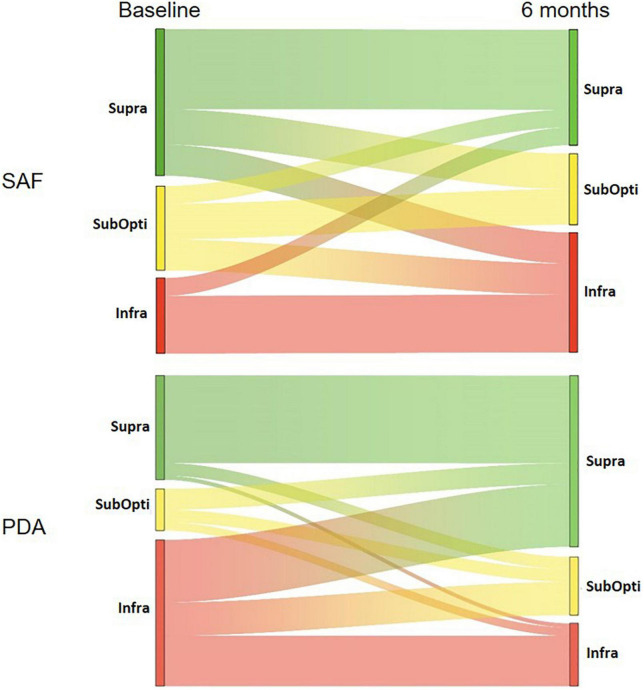
Sankey diagrams showing the interclass evolution of patient according to the ANSES 2016 recommendations. Patients were classified into InfraRDA, SubOptiRDA or SupraRDA at baseline and 6 months. Interclass evolution during follow-up is depicted in SAF and PDA groups. For each class, the height of bars corresponds to the number of patients (see Table 4).

Considering only the InfraRDA patients at baseline, there was no significant difference in the median calcium intake between the two groups (578 mg/day for SAF and 583 mg/day for PDA, *p* = 0.69, Wilcoxon–Mann–Whitney) (Table 2). At 6 months, we observed a median increase of 66 mg of calcium intake per day in the SAF group and of 358 mg in the PDA group (*p* = 0.0082; Wilcoxon Mann–Whitney) (Table 3). To examine interclass evolution of patients during follow-up, we reclassified the entire population according to ANSES 2016 recommendations at 6 months. Among the 35 InfraRDA patients at baseline, only 12 patients (34%) remained in the InfraRDA class after 6 months of follow-up in the PDA group, compared to 13 patients of 17 (76%) in SAF group (Table 4 and Figure 3). Moreover, the number of InfraRDA patients increased in the SAF group (29 vs. 17 patients at baseline) while the number of SupraRDA patients decreased (26 vs. 36 patients at baseline). Indeed, 17 out of 36 SupraRDA patients in the SAF group reduced their calcium intake [9 patients (25%) moved to the InfraRDA class and 8 (22%) moved to the SubOptiRDA class] (Table 4 and Figure 3). In the same group, 7 patients in the SubOptiRDA class (43%) moved to the InfraRDA class at the end of the follow-up period (Table 4 and Figure 3).

**TABLE 4:** Evolution of baseline population distribution in the classes after 6 months (number of patients).

			6 months			
			InfraRDA	SubOptiRDA	SupraRDA	Total
Baseline	SAF	InfraRDA	13	0	4	**17**
		SubOptiRDA	7	6	3	**16**
		SupraRDA	9	8	19	**36**
		Total	**29**	**14**	**26**	**69**
	PDA	InfraRDA	12	8	15	**35**
		SubOptiRDA	2	3	5	**10**
		SupraRDA	1	3	21	**25**
		Total	**15**	**14**	**41**	**70**

SAF, standard advice form approach; PDA, personalized dietary advice approach.

### Analysis of calcium intake using the 2019 ANSES guidelines

As the ANSES guidelines changed over the course of the study (30), data were analyzed considering the new recommendations (950 mg/day for adults and 1,000 mg/day for adults under the age of 24 years). We stratified the study population according to these recommendations (Supplementary Table 1). The effect of the new classification produced only minor changes in the distribution of patients between groups. The results of the analyses of the primary objective reported similar results, confirming the efficacy of personalized advice by a dietitian (Supplementary Table 3 and Supplementary Figure 2).

### Evolution of other nutrient intakes and disease outcomes at 6 months

We also evaluated patient intake of other nutrients between groups after 6 months (Table 5 and Figures 2B–D). In the PDA group, proteins, lipids, and vitamin D intakes increased with a median increase of 8 g/day, 5 g/day, and 1 μg/day, respectively. In contrast, the median intake of these nutrients decreased in the SAF group by 9 g/day, 15 g/day, and 0.4 μg/day, respectively (*p* = 0.0007, *p* = 0.032, and *p* < 0.001, respectively). The nutrient increase in the PDA group was associated with global increase in energy intake (+30.5 Kcal/day), and nutrient intake decrease in the SAF group was associated with a strong decrease in energy intake (−258 Kcal/day) (*p* = 0.0277) (Table 5 and Figure 2E). No difference was observed for 25OHD levels between groups at 6 months (Table 5 and Figure 2F). No influence of the intervention was observed on MS outcomes (PASAT, EDSS, HADS, EQ-5D) (data not shown).

**TABLE 5:** Daily nutrients intake evolution over 6 months.

	Nutrient evolution at 6 months				
	SAF		PDA		
Nutrient	**N**	**Median** **IQR** [**q1;q3**]	**N**	**Median** **IQR** [**q1;q3**]	***P*-value**
Protein, g/day	69	−9 [−26; 4]	70	8 [−11; 20]	**0**.**0007**
Lipids,g/day	69	−15 [−25; 6]	70	5 [−26; 29]	**0**.**0321**
Carbohydrates, g/day	69	−13 [−53; 15]	70	−4 [−64; 39]	0.5261
Energy, Kcal/day	69	−258 [−495; 55]	70	30.5 [−413; 427]	**0**.**0277**
Vitamin D, μg/day	69	−0.4 [−1; 0]	70	1 [0; 2.7]	**<0**.**0001**
Serum 25OHD, nmol/L	64	−5 [−18; 12]	69	−8 [−21; 15]	0.6076

Data presented as median change [q1; q3]. *P*-values in bold denote significant differences. Statistical significance between groups was calculated by Wilcoxon–Mann–Whitney test. SAF, standard advice form approach; PDA, personalized dietary advice approach.

### Disease effects on calcium intake

Because MS is associated with cognitive disorders, neurological disability, altered quality of life and mood disorders that may affect the understanding and practical implementation of dietary recommendations, we analyzed the effect of these conditions on calcium intake evolution between groups (Supplementary Table 2). We compared neurological disability (EDSS score), cognitive status (PASAT), anxiety (HADS A), depression (HADS D), and quality of life (EQ-5D) in both groups at baseline and at 6 months. Analysis of evolution of calcium intake in each group according to these conditions showed that the efficacy of PDA was still significant in the population with no or low disability (EDSS ≤ 4) (*q*-value = 0.0002) and population with low or high cognitive status (low < 39 answers < high) (*q*-value = 0.0002 and 0.0009, respectively) (Supplementary Table 2). We observed similar results for anxiety and depression independently of the level of symptoms (low or serious) and for the quality of life (good or bad) (Supplementary Table 2).

We also analyzed how changes in diet after MS diagnosis, as an indirect effect of MS, could affect calcium intake, as some patients reduce the consumption of dairy products and gluten after diagnosis. We observed that 24% of the patients included in this study changed their eating habits because of MS diagnosis. Of note, the majority of participant declared not following any restrictive or specific diet [89.6% (SAF) and 87.8% (PDA)], while very few patients (<3%) declared following a vegetarian or a gluten free diet in the SAF and in the PDA groups. However, no difference was observed between the median calcium intake at inclusion of patients who had not changed their diet (871 mg/day) and those who had changed their diet (840 mg/day) (*p* = 0.8385, Supplementary Table 4). Interestingly, 34 patients (19%) of patients avoided lactose at baseline while only one patient avoided lactose at 6 months.

### Influence of education level on calcium intake

**FIGURE 4:**
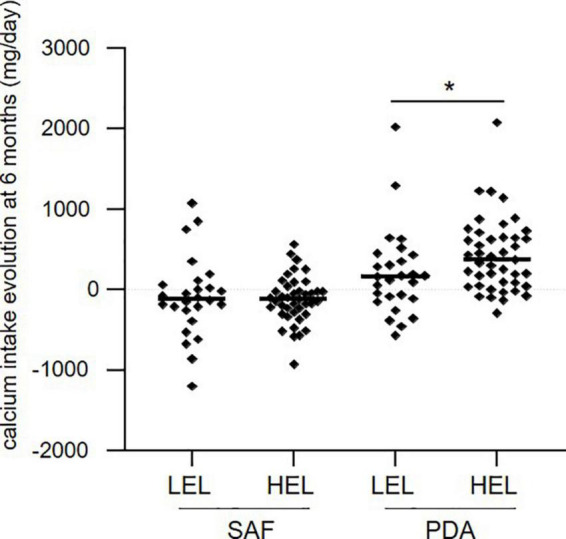
Evolution of calcium intake at 6 months in each group, according to the level of education. LEL, low education level; HEL, high education level. Means are shown as horizontal bars. Statistical significance was determined using the Wilcoxon–Mann–Whitney test. *p < 0.05.

We examined the influence of education level on the evolution of calcium intake after 6 months. Patients in the PDA group with high education level (HEL) had a greater median increase of calcium intake after 6 months (373 mg/day) compared to patients with a low education level (LEL) (159 mg/day) (*p* = 0.023, Figure 4), while in the SAF group, the education level did not affect the evolution of calcium intake over 6 months (−115.5 mg/day for LEL and −120 mg/day for HEL, *p* = 0.87, Figure 4). These results strongly suggest that education level affects the effectiveness of the PDA approach.

## Discussion

In this study, we show that personalized advice by a dietitian appears to be more effective than the delivery of written recommendations to increase daily calcium intake in MS patients. Indeed, MS patients with insufficient calcium intake (InfraRDA) in the PDA group showed a significant increase in their calcium intake as compared to those from the SAF group. This positive effect of personalized dietary management is confirmed in the SubOptiRDA class in which 50% of patients moved to the SupraRDA in the PDA group and only 19% in the SAF group.

In addition, given that low BMI is a risk factor of osteoporosis, the increase of other nutrients intake and energy intake in the PDA group could constitute a complementary beneficial effect of the PDA approach (37). Moreover, efficient increase in calcium intake based on diet improvement could minimize the need for calcium supplementation potentially at risk of lithiasis (38). Here, very few patients used calcium supplementation and no SAE was observed, especially in the SupraRDA class in the PDA group, suggesting that increasing calcium intake by dietary means might be preferable to calcium supplementation. Interestingly, despite a significant increase of dietary vitamin D intakes between baseline and 6 months in the PDA group, our study population had 25OHD levels below the recommended concentration range of 75–150 nmol/L, suggesting that vitamin D supplementation might still be necessary in MS patients to sustain or improve BMD as these patients show low bone mass, even in early-stage disease (9–11, 39). In this case, vitamin D supplementation needs to be controlled as severe calcium deficiency and excessive vitamin D supplementation may lead to bone density loss and osteoporosis (39).

Looking at factors influencing the PDA efficacy, we showed that efficacy was not affected by age, level of disability or cognitive status, suggesting that this approach would not be altered by the severity of the disease and may be suitable for all MS patients. These results may also suggest that PDA could constitute a more effective approach than SAF in the population at high risk of osteoporosis.

In our study the median calcium intake of the SAF group decreased significantly at 6 months compared to inclusion (*p* = 0.025), suggesting that the written recommendations may be misinterpreted by MS patients, leading to a paradoxical negative effect. This effect could be the consequence of the disease and/or the GRIO form. MS is associated with cognitive disorders, neurological disability, altered quality of life and mood disorders that may affect the understanding and practical implementation of written dietary recommendations. The GRIO form only provides information on daily-recommended calcium intake and calcium content from enriched food and water. This information seems to be insufficient for MS patients to change their eating habits and might also explain why in the SAF group we did not observe an increase of other nutrients (proteins, lipids, and vitamin D) after 6 months. Long-term follow-up would be necessary to confirm this effect.

Our results highlight the importance of a personalized nutrition advice program as compared to general written recommendations. Several studies have tested the value of nutritional programs in increasing calcium intake to prevent osteoporosis. A study from Beaudoin et al. on 1175 women compared two nutritional education programs (40). One program provided written recommendations (WM) and the other written recommendations and video support explaining the recommendations in detail (VC). Results showed that in women over the age of 50 years, the video program was not more effective than written recommendations alone. Median calcium intake increased by 91 mg/day in the WM group and 93mg/day in the VC group, showing the positive effect of written recommendations in increasing calcium intake in osteoporotic patients. However, another study (41) involving 80 younger participants (19–29 years) that used a 45-min slide show at inclusion and then 8 weeks later, showed that the mean calcium intake of these patients decreased slightly from 961.3 to 905.0 mg/day, demonstrating the lack of effectiveness of this approach. In a study conducted by Chan et al., 20 women followed a nutritional educational program consisting of a nurse delivering personalized advice accompanied by phone call for follow-up (42). This program changed the eating habits of participants and significantly increased their consumption of dairy products, contrary to the situation for participants receiving no advice. However, the authors did not analyze the calcium intake of participants. Finally, a study on Chinese female immigrants in the United States suffering from severe calcium deficiencies showed that personalized and interactive nutritional workshops resulted in a significant increase in calcium intake as compared to the control group with no dietary intervention (43). The increase achieved in this study was comparable to the results obtained in our study (−26.3 mg/day in the control group vs. +213.2 mg/day in the intervention group), although these patients remained with insufficient intake despite changing their diet.

All these studies suggest the effectiveness of personalized nutritional programs, although less efficiently than in our study. It is worth noting that the above studies were carried out on female populations and, although the majority of participants in our study were women, it also included male individuals (143 women and 39 men).

Other studies have also investigated the impact of dietary advice in other diseases such as cancer. One study investigated the effect of individual dietary advice versus protein-enriched nutritional supplements on nutrition, morbidity and quality of life in colorectal cancer patients three months after undergoing radiotherapy, showing that both interventions had beneficial effects on health during radiotherapy (44). However, only dietary advice was able to maintain these positive effects three months after radiotherapy. Similarly, Insering et al. reported that in cancer patients undergoing radiotherapy, personalized dietary advice resulted in better nutritional intake than written and general recommendations (45). These studies therefore suggest that even in a context of severe disease, the use of dietary advice is probably more effective by promoting better collaboration with the patient and appropriate support.

Inadequate diets play a major role in the increasing prevalence of malnutrition in all its forms and can even become a risk factor for osteoporosis or other diseases (46, 47). In our study, 24% of patients reported changing their eating habits due to MS, but we did not observe any significant difference in their median calcium intake at baseline as compared to the other patients of the study, suggesting that diet changes instigated by these patients did not impact calcium intake.

Education level is a socio-economic factor that influences dietary pattern. Several studies have shown that unhealthy diets are observed more often in people of lower socioeconomic status, based on education level, income level, and type of occupation (21–23, 48). In our study, the PDA approach was less effective in patients with low education level than for those with high education level. This effect could be explained by the fact that this population is less compliant with nutritional recommendations or dietary guidelines (22, 48). Longer follow-up may be necessary to markedly change eating habits in this population.

Our study has some limitations. Despite randomization, the difference in basal calcium intake between the study groups was significant, probably contributing to the negative effect of SAF on calcium intake evolution in this group. Although we perform an analysis with adjusted linear model on baseline calcium intake and a *post-hoc* analysis based on the analysis of patients with insufficient or suboptimal calcium intake at baseline, this cannot replace a stratified random sampling on baseline calcium intake that would have been much preferable. In our study, almost one quarter of patients did not attend all the visits, resulting in a limited power of statistical analyses. The impact of this factor was probably limited since these patients were spread equally between both groups.

## Conclusion

This work shows for the first time the importance of the effect of a three-month dietary management program based on personalized interviews in improving calcium intakes in MS patients. Although, long term follow-up coupled to BMD assessment would be necessary to demonstrate a positive effect on osteoporosis prevention in MS patient. Early systematic screening for calcium deficiencies in MS patients seems to be an interesting and promising approach. The safety of dietary management appears to be excellent in view of the total absence of serious adverse events linked to the PDA approach. In addition, it minimizes drug consumption and the risks of iatrogenesis. More generally, due to the potential benefits of nutritional education on the overall health of patients, dietary management might be proposed to integrate the general therapeutic education program in these patients.

## Data availability statement

The raw data supporting the conclusions of this article will be made available by the authors, without undue reservation.

## Ethics statement

The studies involving human participants were reviewed and approved by Persons Protection Committee (CPP) Sud Méditerranée III (# 2015.11.01 ter). The patients/participants provided their written informed consent to participate in this study.

## Author contributions

ET and SF: conceptualization. TC: methodology. SF, ET, GC, PP, ES, VV, and CB: investigation. SL-C and SA: statistical analysis supervision-validation. ET, HA, SL-C, and SF: data analysis and interpretation. ET and HA: writing—original draft preparation. ET, HA, EE, SL-C, and DR: writing—review and editing. All authors have read and agreed to the published version of the manuscript.
