# Supplementary material for: Personalized dietary advices provided by a dietitian increase calcium intake in outpatients with multiple sclerosis—Results from a randomized, controlled, single-blind trial
Source: Front Nutr. 2023 Jan 17;9:919336. doi: 10.3389/fnut.2022.919336 (PMC9887148; doi:10.3389/fnut.2022.919336)
Supplement: Supplementary file 1 [file Data_Sheet_1.docx]

**Supplementary Material:**

**Supplementary figures legends:**

Supplementary Figure 1: GRIO form. Advice form from GRIO (Groupe de Recherche et d’Information sur les Ostéoporoses) received by patient in the SAF group. The GRIO form is a document in French, composed of two pages indicating the optimal target for calcium intake and providing information about calcium content of different fruits, vegetables, mineral waters and other protein-rich food. It has been elaborated by the French group of research and information on osteoporosis (GRIO) and is available on the GRIO’s website (http://www.grio.org/).

Supplementary Figure 2: Sankey diagrams showing the interclass evolution of patient according to the ANSES 2019 recommendations. Patients were classified into InfraRDA, SubOptiRDA or SupraRDA at baseline and 6 months. Interclass evolution during follow-up is depicted in SAF and PDA groups. For each class, the height of bars corresponds to the number of patients (see Supplementary Table 3).

Supplementary Table 1: Study population stratification according to 2016 and 2019 ANSES recommendations.

|  |  | InfraRDA | SubOptiRDA | SupraRDA | ANSES recommendations |
| --- | --- | --- | --- | --- | --- |
| 2016 ANSES recommendations for Daily calcium intake (mg/day) | men over 65 years old and postmenopausal women aged over 51 years old | < 900 | 900 to 1200 | >1200 | 1200 |
|  | others adults | < 750 | 750 to 900 | >900 | 900 |
| 2019 ANSES recommendations for Daily calcium intake (mg/day) | adults < 24 years old | < 750 | 750 to 1000 | >1000 | 1000 |
|  | others adults | < 750 | 750 to 950 | >950 | 950 |

Supplementary Table 2: Comparison of calcium intake evolution between SAF and PDA groups according to physical disability, mental health and quality of life.^1^

|  | **Wilcoxon Scores (Rank Sums)** | | | | |
| --- | --- | --- | --- | --- | --- |
|  |  |  | **N** | **Mean** | **q-value** |
| **EDSS** | **No or low disability** | SAF | 61 | -138 | **0.0002** |
|  |  | PDA | 64 | 214 |  |
|  | **High disability** | SAF | 8 | -20 | **0.0197** |
|  |  | PDA | 6 | 299.5 |  |
| **PASAT** | **Low cognitive status** | SAF | 32 | -168 | **0.0002** |
|  |  | PDA | 30 | 241.5 |  |
|  | **High cognitive status** | SAF | 28 | -134.5 | **0.0009** |
|  |  | PDA | 32 | 199.5 |  |
| **HADSa** | **No or low anxiety disorder** | SAF | 48 | -117 | **0.0002** |
|  |  | PDA | 48 | 241.5 |  |
|  | **serious anxiety disorder** | SAF | 21 | -120 | **0.0195** |
|  |  | PDA | 18 | 195 |  |
| **HADSd** | **No or low depressive disorder** | SAF | 64 | -125.5 | **0.0002** |
|  |  | PDA | 63 | 203 |  |
|  | **serious depressive disorder** | SAF | 5 | 28 | 0.1421 |
|  |  | PDA | 5 | 712 |  |
| **EQ5D** | **Bad quality of life** | SAF | 32 | -118 | **0.0002** |
|  |  | PDA | 34 | 280 |  |
|  | **good quality of life** | SAF | 36 | -120 | **0.0002** |
|  |  | PDA | 35 | 230.5 |  |

Statistical significance between groups was calculated by Wilcoxon-Mann-Whitney test and Holm and false discovery rate (FDR) correction were used for multiple comparisons. q-values in bold denote significant differences.

SAF: standard advice form approach; PDA: personalized dietary advice approach; EDSS: Expanded Disability Status Scale: PASAT: The Paced Auditory Serial Addition Test; HADS: Hospital Anxiety and Depression Scale; EQ5D: 5 dimensions scoring descriptive system for quality of life.

Supplementary Table 3: Evolution of baseline population distribution in the classes after 6 months (stratification according to ANSES recommendations 2019).

|  |  |  | at 6 months | | |  |
| --- | --- | --- | --- | --- | --- | --- |
|  |  |  | **InfraRDA** | **SubOptiRDA** | **SupraRDA** | **Total** |
| Baseline | SAF | **InfraRDA** | 13 | 0 | 4 | **17** |
|  |  | **SubOptiRDA** | 7 | 8 | 4 | **19** |
|  |  | **SupraRDA** | 7 | 8 | 18 | **33** |
|  |  | **Total** | **27** | **16** | **26** | **69** |
|  | PAD | **InfraRDA** | 8 | 10 | 16 | **34** |
|  |  | **SubOptiRDA** | 3 | 5 | 11 | **19** |
|  |  | **SupraRDA** | 0 | 3 | 14 | **17** |
|  |  | **Total** | **11** | **18** | **41** | **70** |

SAF: standard advice form approach; PDA: personalized dietary advice approach.

Supplementary Table 4: Calcium intake and prevalence of dietary change in study population after MS diagnosis (p=0.8385, Wilcoxon Mann Whitney).

|  | **Change in dietary since diagnosis** | | |
| --- | --- | --- | --- |
|  | **No** | **Yes** | **Total** |
| N | 109 | 29 | 138 |
| Median | **871** | **840** | 852 |
| IQR [q1;q3] | [619; 1132] | [622; 1058] | [619; 1097] |
