# Supplementary figures and images for: Personalized dietary advices provided by a dietitian increase calcium intake in outpatients with multiple sclerosis—Results from a randomized, controlled, single-blind trial
Source: Front Nutr. 2023 Jan 17;9:919336. doi: 10.3389/fnut.2022.919336 (PMC9887148; doi:10.3389/fnut.2022.919336)

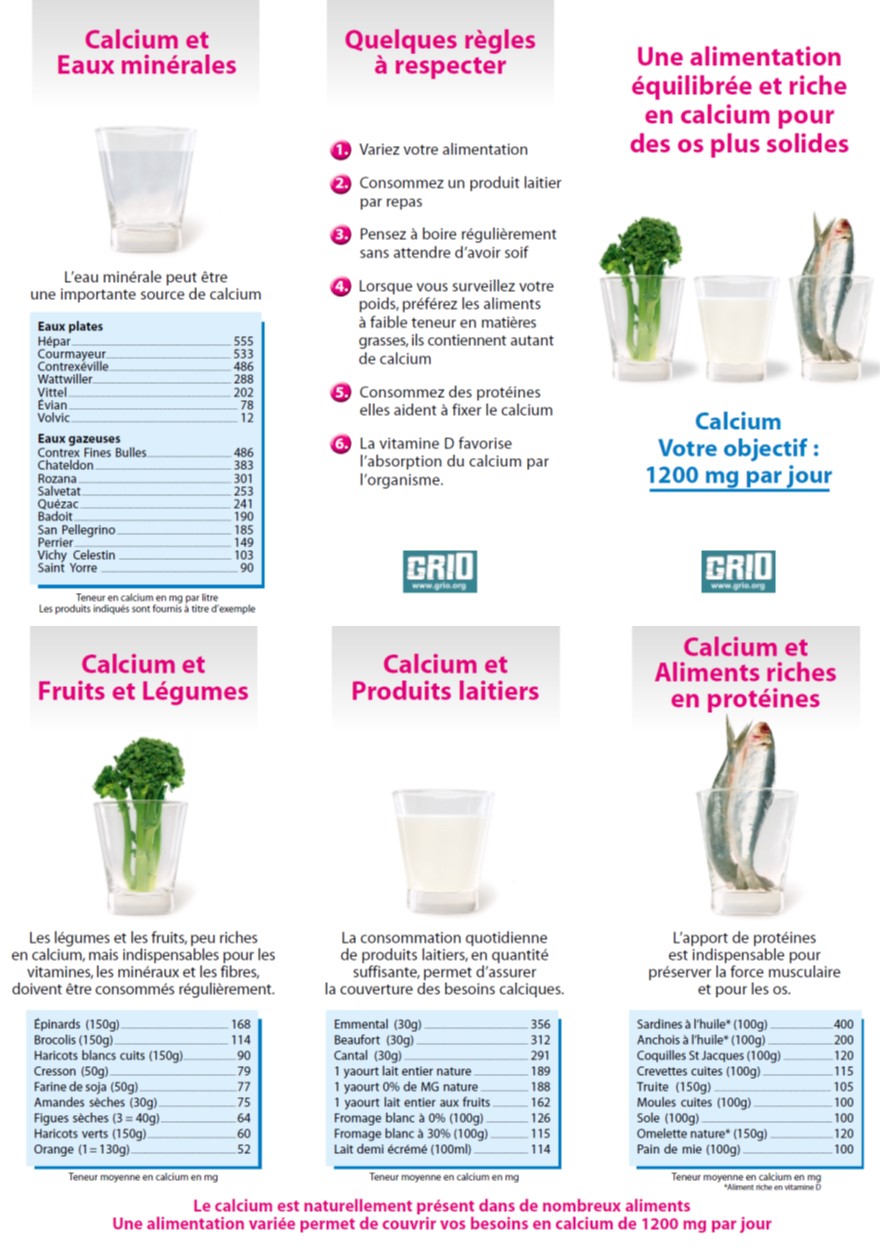

Supplement: Supplementary file 2 [file Image_1.jpeg]

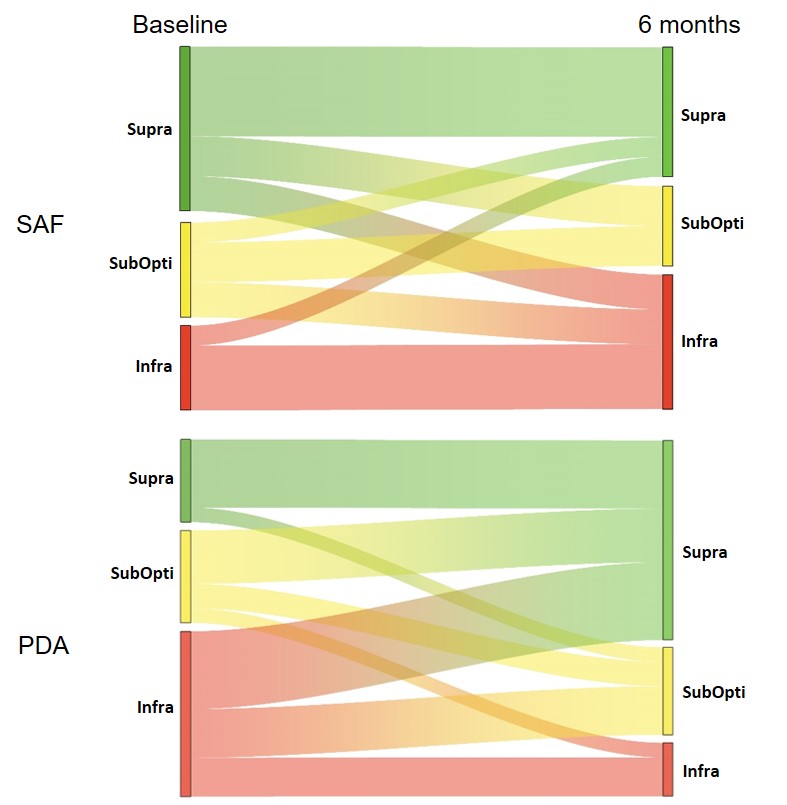

Supplement: Supplementary file 3 [file Image_2.jpeg]
